# MicroRNAs as New Biomarkers for Diagnosis and Prognosis, and as Potential Therapeutic Targets in Acute Myeloid Leukemia

**DOI:** 10.3390/ijms19020460

**Published:** 2018-02-03

**Authors:** Stefania Trino, Daniela Lamorte, Antonella Caivano, Ilaria Laurenzana, Daniela Tagliaferri, Geppino Falco, Luigi Del Vecchio, Pellegrino Musto, Luciana De Luca

**Affiliations:** 1 Laboratory of Preclinical and Translational Research, IRCCS-Referral Cancer Center of Basilicata (CROB), 85028 Rionero in Vulture, Italy; stefania.trino@gmail.com (S.T.); daniela.lamorte@crob.it (D.L.); antonella.caivano@crob.it (A.C.); ilaria.laurenzana@crob.it (I.L.); 2 Biogem Scarl, Istituto di Ricerche Genetiche ‘Gaetano Salvatore’, 83031 Ariano Irpino, Italy; daniela.tagliaferri11@gmail.com (D.T.); geppino.falco@unina.it (G.F.); 3 Department of Biology, University of Naples Federico II, 80147 Naples, Italy; 4 CEINGE Biotecnologie Avanzate s.c.a r.l., 80147 Naples, Italy; luigi.delvecchio@unina.it; 5 Department of Molecular Medicine and Medical Biotechnologies, University of Naples Federico II, 80138 Naples, Italy; 6 Scientific Direction, IRCCS—Referral Cancer Center of Basilicata (CROB), 85028 Rionero in Vulture, Potenza, Italy; pellegrino.musto@crob.it

**Keywords:** microRNAs, acute myeloid leukemia, biomarkers, therapeutic targets

## Abstract

Acute myeloid leukemias (AML) are clonal disorders of hematopoietic progenitor cells which are characterized by relevant heterogeneity in terms of phenotypic, genotypic, and clinical features. Among the genetic aberrations that control disease development there are microRNAs (miRNAs). miRNAs are small non-coding RNAs that regulate, at post-transcriptional level, translation and stability of mRNAs. It is now established that deregulated miRNA expression is a prominent feature in AML. Functional studies have shown that miRNAs play an important role in AML pathogenesis and miRNA expression signatures are associated with chemotherapy response and clinical outcome. In this review we summarized miRNA signature in AML with different cytogenetic, molecular and clinical characteristics. Moreover, we reviewed the miRNA regulatory network in AML pathogenesis and we discussed the potential use of cellular and circulating miRNAs as biomarkers for diagnosis and prognosis and as therapeutic targets.

## 1. Introduction

Acute myeloid leukemia (AML) is an aggressive hematological malignancy characterized by the abnormal proliferation and differentiation of myeloid progenitor cells [1,2]. This neoplasm is the most common acute leukemia in adult patients and can arise “de novo” or as a secondary event [3]. In particular, secondary AML can derive from a previous clonal disorder of hematopoiesis, such as myelodysplastic syndrome (MDS) or chronic myeloproliferative neoplasm (MPN), or following a previous therapy (chemotherapy or radiotherapy, immunosuppressive drugs or environmental leukemogenic agents) [4,5]. These myeloid malignancies are characterized by acquired common recurrent mutations in hematopoietic progenitors. Gene alterations can contribute to hematopoietic transformation conferring a growth advantage or modifying the expression of key transcriptional targets in myelopoiesis. These mutations occur in genes with known roles in regulating chromatin and/or methylation states in hematopoietic progenitors (e.g., mixed-lineage leukemia (MLL)), or exert a role in altering epigenetic pattern in myeloid malignancies (e.g., TET methylcytosine dioxygenase 2 (TET2), DNA methyltransferase 3A (DNMT3A), sex combs-like 1 (ASXL1), isocitrate dehydrogenase (IDH) 1 and IDH2). They contribute to the pathogenesis, and could represent attractive targets for directed therapy in myeloid malignancies [6].

AML is a highly heterogeneous disease at molecular and clinical levels; well-identified genetic and cytogenetic aberrations hold a pathogenetic and prognostic relevance in this neoplasm [3]. The new 2016 WHO classification defined six major AML entities incorporating genetic alterations with morphology, immunophenotype, and clinical presentation: (i) AML with recurrent genetic abnormalities; (ii) AML with myelodysplasia-related changes; (iii) therapy-related AML; (iv) AML not otherwise specified; (v) myeloid sarcoma; (vi) myeloid proliferation related to Down syndrome [7]. On the basis of cytogenetic and molecular profile, AML patients can be divided into favorable, intermediate, and adverse prognostic risk groups [3]. AML treatment is currently based on high-intensity induction chemotherapy in eligible patients to achieve complete remission (CR), followed by consolidation regimen, including conventional chemotherapy, as well as hematopoietic cell transplantation [1]. However, patients achieving CR can relapse due to the persistence of minimal residual disease (MRD) [1,8].

It is important to underline, beyond, that AML is perpetuated and maintained by a small population of leukemia initiating cells or leukemia stem cells (LSCs) [9,10]. These cells exhibit similar characteristics of hematopoietic stem cells (HSCs), including the ability to generate identical daughter cells as well as differentiated cells. On the contrary, LSCs have different properties than the AML bulk population [11,12]. These features make LSCs difficult to eliminate with standard chemotherapy, and therefore, result in a source of malignancy resistance and relapse.

MicroRNAs (miRNAs) are evolutionary conserved short non-coding single-stranded RNAs (19–22 nucleotides) [13,14] that negatively regulate mRNA stability [14,15,16]. They play an essential role in many biological functions, such as cell growth, proliferation, differentiation, and apoptosis [13,17]. Moreover, miRNAs can act as oncogenes or tumor suppressors, contributing to malignant transformation in solid and hematological tumors, including AML [16,18,19]. In addition, miRNAs regulate different mRNA targets, and their modulation can represent a potential therapeutic target in leukemic progenitors, as well as in LSCs. In this context, the identification of miRNA expression patterns in HSCs, LSCs, and leukemic progenitors is required.

miRNA expression is frequently deregulated in AML by different mechanisms, like: (i) copy number alterations; (ii) epigenetic changes; (iii) miRNA location in proximity of oncogenic genomic region due to chromosomal translocation or overexpression of protein-coding gene; (iv) aberrant targeting of miRNA promoter regions by altered transcription factors or oncoproteins; and finally (v) deregulated miRNA processing [14].

During the last 15 years, microarray was a widely used platform to investigate miRNA expression in AML. In particular, many studies identified the expression signatures associated with different AML subtypes, especially with specific molecular and cytogenetic alterations [20]. Recently, the emerging RNA-Sequencing technologies were used to evaluate miRNA abundance and to discover new miRNAs in AML [21].

In this review, we summarized miRNAs differentially expressed in AML subtypes, LSCs, and HSCs, their role in AML pathogenesis, their potential use as disease biomarkers for diagnosis/prognosis and as therapeutic targets.

## 2. Distinctive Pattern of miRNAs in AML

Molecular and cytogenetic features are currently defined as entities of clinical significance, and are included in diagnostic criteria of AML, according to 2016 WHO classification [7].

Numerous studies demonstrated the existence of distinct miRNA profiles in different AML subtypes, indicating that miRNA signature contributed to AML heterogeneity, and suggesting its potential inclusion in clinical setting [22,23,24,25,26,27,28,29,30,31,32,33,34,35,36,37,38,39,40,41,42,43]. In Table 1 were reported all major studies comparing miRNA profile between AML blasts and normal cells, and among AML with recurrent genetic abnormalities [22,23,24,25,26,27,28,29,30,31,32,33,34,35,36,37,38,39,40,41,42,43]. Most of the reported studies exhibited high concordance in AML miRNA profile. For example, in patients with NPM1 mutation, different authors found the upregulation of *miR-10a*, *miR-10b*, and *miR-196b* [26,37,38], and the downregulation of *miR-192* [36,38]. A common signature was also reported in patients with t(8;21) showing the upregulation of *miR-126* [22,23] and *miR-146a* [24,25], while overexpression of *miR-155* was strongly associated with FLT3-internal tandem duplication (ITD) alteration [26,34,36,39].

Therefore, some studies showed partial overlap in miRNA profile, and this is probably due to different microarray platforms, sample type, or comparison analysis among AML subtypes.

According to cytogenetic and molecular alterations, European LeukemiaNet divided AML patients into three risk groups: favorable, intermediate, or adverse [44]. Each subtype seems to exhibit a unique miRNA signature that distinguishes it from other AML subtypes. For example, Marcucci G et al. reported the increased expression of all members of *miR-181* family in patients with favorable risk (cytogenetically normal AML (CN-AML) with CEBPA mutations) [40] and its decreased expression in a high risk subgroup (CN-AML with FLT3-ITD and/or wild-type NPM1) [45]. Similarly, in other studies, *miR-181* was also observed increased in cytogenetically abnormal AML (CA-AML) with favorable cytogenetic abnormalities, such as t(15;17) (favorable risk), and decreased in CA-AML with unfavorable cytogenetic alterations, such as MLL-rearrangements (adverse risk) [23,46]. Garzon et al. compared CN-AML with CA-AML patients, identifying a signature in CN-AML of 10 upregulated *(miR-10a*, *miR-10b*, *miR-16-2*, *miR-21*, *miR-26a*, *miR-30c*, *miR-181b*, *miR-192*, *miR-368*, and *let-7a-2*) and 13 downregulated miRNAs (*miR-126*, *miR-145*, *miR-182*, *miR-183*, *miR-191*, *miR-193*, *miR-194*, *miR-196b*, *miR-199a*, *miR-200c*, *miR-203*, *miR-204*, and *miR-299*) [34]. *miR-139-5p* was found downregulated in CN-AML with mutated FLT3, and acted as tumor suppressor in a primary AML transplant model [47]. Dixon-McIver et al. reported that the expression of *miR-9* and *let7b* was low in the favorable groups, and high in intermediate and adverse AML groups [24]. They also found *miR-125b* mainly expressed in AML with a normal karyotype [24].

miRNA expression was also correlated with bone marrow (BM) morphology. Chen et al. demonstrated that mature *miR-181* expression was detectable in BM undifferentiated progenitor cells [48]. Moreover, its expression was strongly correlated with the AML morphological subtype, resulting in elevated levels in samples with M1 or M2 French–American–British (FAB) classification, compared with the samples with M4 or M5 morphology [49]. By contrast, in normal BM, *miR-181a* has been reported to be preferentially expressed in B cells, T cells, monocytes, and granulocytes, which are more closely allied to M4 and M5 subtypes [50]. Another study, instead, compared M1 with M5 miRNA profile, indicating the higher expression of not only *miR-181a* and *miR-181b*, but also miR-181a*, *miR-181d*, *miR130a*, *miR-135b*, *miR-146a*, *miR-146b*, and *miR-663* in FAB M1. Instead, *miR-21*, *miR-193a*, and *miR-370* were overexpressed in FAB M5 samples [51]. Recently, de Leeuw et al. identified the highest expression of *miR-551b* in the most primitive cell populations, the HSC and multipotent progenitors; instead, differentiated progenitors, monocytes and lymphocytes, showed decreased and absent *miR-551b* expression, respectively. This expression pattern might indicate a *miR-551b* role in early hematopoiesis and stem cells. Therefore, authors hypothesized that its expression in AML might be indicative of an immature leukemia with stem cell features. In fact, they found a high *miR-551b* expression in undifferentiated AML (FAB M0); on the contrary, *miR-551b* was not expressed in patients with favorable cytogenetics [52].

Yang et al. demonstrated that *miR-122* was downregulated in AML with respect to normal controls, and its decreased expression was more frequently observed in FAB M7 with respect to M1–M6 subtypes, and in unfavorable cytogenetic abnormalities, acting as tumor suppressor [53].

In a recent paper, we evaluated *miR-128a* expression in AML patients stratified for morphologic features, observing high expression levels of this miRNA in AML with maturation and in acute promyelocytic leukemia cases compared to healthy subject CD34^+^ [31].

In any case, the majority of reported studies, based on the FAB classification system, needs to be revised according to the 2016 WHO classification [7].

Thus, the all cited literature data highlighted that the aberrant expression of miRNAs contributes to AML heterogeneity.

## 3. miRNAs Involved in AML Pathogenesis

MicroRNAs alterations are recognized, through various mechanisms, to be involved in AML pathogenesis [54]. In particular, literature data indicate that miRNAs can induce leukemogenesis, altering numerous biological processes, including self-renewal, survival, proliferation, differentiation, and epigenetic regulation. Their involvement in leukemic development and progression is due to their collaboration with deregulated protein (oncogene or tumor suppressor), either by direct control of protein translation, or by working jointly with them to promote malignancy [14,54].

To summarized these concepts, we have illustrated in Table 2 the most important miRNAs found to play a role in AML, specifying for each miRNA the altered expression (up or downregulation), the mechanisms of dysregulation, their targets, and their functional effects in AML pathogenesis [23,28,31,32,33,41,55,56,57,58,59,60,61,62,63,64,65,66,67,68,69,70,71,72,73,74,75,76,77,78,79,80,81,82,83,84,85,86,87,88,89,90,91,92,93,94,95,96,97,98,99,100,101,102,103,104,105]. However, literature data revealed that miRNA dysregulation in AML can have different effects on the basis of their expression levels. *miR-155*, for example, is upregulated respect to healthy subjects having an oncogenic function in AML FLT3-ITD subgroup [91,95]; by contrast, in different AML subsets, it was demonstrated to have a tumor suppress function [106,107].

Narayan et al. demonstrated in three different murine models of AML (HoxA9/Meis1, MLL-ENL, MLL-AF9) that this is due to different expression levels of *miR-155*. High doses of this miRNA repressed clonal proliferation, instead, intermediate levels of *miR-155* showed an oncogenic function, leading to an increased proliferation and enhancing colony formation [108]. Moreover, this group confirmed that the intermediate *miR-155* expression level was associated with poor prognosis in pediatric AML patients, suggesting a prevalent oncogenic function in this hematological neoplasm [34,95,108].

miRNAs exert a functional role in leukemogenesis both in vivo and in vitro controlling their mRNA targets. Generally, miRNA–mRNA relationships are related to AML subtypes, and some targets play an important role than others, depending on the AML cytogenetic and molecular signatures [109], as reported in Table 2.

One of the most studied miRNAs in AML, *miR-155*, for example, negatively regulated the transcription factor PU.1, involved in hematopoietic cell determination [110]. Basova et al. confirmed this data in AML pathogenesis, and reported *miR-155* cooperation with Myb. This last acted as an activator of cell proliferation and inhibitor of myeloid maturation, with a hierarchical order of Myb/*miR-155*/PU.1. Moreover, p53 regulated this axis. Its loss caused the upregulation of the two oncogenes Myb and *miR-155*, concomitantly with progressive PU.1 downregulation and characterized aggressive AML. The inhibition of the oncogenes Myb and *miR-155*, or by rescue of PU.1, led to leukemia blast differentiation [111]. *miR-155* was able to induce proliferation of myeloid cells, causing a myeloproliferative syndrome after ectopic expression in myeloid progenitors [95]. Ghani et al. suggested that PU.1 activated *miR-155* expression inducing quiescent HSC to entry into cell cycle, and thus activated their initial differentiation into a transient myeloid progenitor stage. When PU.1 was removed from its binding site at *pre–miR-155*, the lack of expression of *miR-155* blocked proliferation, thus preventing leukemia, and initiated terminal myeloid maturation. Thus, PU.1 was needed to initiate the expression of this miRNA, but not for its maintenance [112].

*miR-17* polycistronic miRNA (*miR-17-92* cluster) has been shown to be highly expressed in human MLL-rearranged leukemia [58,59]. Recently, Wong et al. demonstrated that the cyclin-dependent kinase inhibitor p21 was a critical downstream target of this microRNA. Leukemic cells expressing increased levels of *miR-17* polycistron displayed a higher frequency of LSCs, a block of differentiation, and increased proliferation associated with reduced expression of p21. Moreover, c-myc was shown to be a critical upstream regulator of *miR-17* expression in MLL transformation [58]. Notably, downregulation of the *miR-17p-92* cluster can promote myeloid lineage fate in normal cord blood CD34^+^ HPCs [113].

*MiR-125b* was found upregulated in AML. Chaudhuri et al. demonstrated that *miR-125b* overexpression led to uncontrolled generation of myeloid progenitors and mature myeloid cells that subsequently caused a highly invasive myeloid leukemia. The authors also showed that *Lin28A*, an induced pluripotent stem cell gene, was a bona fide primary target of *miR-125b* in hematopoietic cells. Lin28A downregulation mimicked the preleukemic state induced by *miR-125b*. [74]. *MiR-125b* physiologically regulated hematopoiesis [114]. Studies of miRNA expression profiles in multiple hematopoietic subpopulations demonstrated that *miR-125b* was one of the most expressed miRNAs in HSCs, compared with all other progenitor populations. Moreover, *miR-125b* was specifically enriched in the long-term HSC population [76]. While its level significantly decreased in committed progenitors, *miR-125b* was more highly expressed in common lymphoid progenitors than in common myeloid progenitors [115].

It is known that leukemic cells release extracellular vesicles (EVs) that modify target cells in BM microenvironments by transferring their metabolic, protein, and genetic content, such as mRNA, DNA, and miRNA [116,117]. EV miRNAs act to create a favorable BM microenvironment that sustains and support leukemia [117,118,119]. In this context, Hornick et al. showed a direct effect of AML exosome (Exo) miRNAs compromising hematopoiesis of healthy hematopoietic stem and progenitor cells (HSPCs) [120]. This study suggested that leukemia-derived Exo were sufficient to induce systemic impairment of hematopoiesis, demonstrating that *miR-150* and *miR-155* and their regulatory network were sufficient to suppress HSPC clonogenicity. [120]. Similarly, Razmkhah et al. demonstrated that AML BM microvesicles (MVs) induced colony formation and increased expression of two leukemic oncomiRs, *miR-21* and *miR-29a*, in healthy HSPCs [121]. In addition, Horiguchi et al. revealed that *miR-7977* in EVs from AML/myelodysplastic syndrome induced a reduction of hematopoietic-supportive capacity of normal BM mesenchymal stem cells [122].

## 4. Differential Expression of MiRNAs in HSCs and LSCs

It is well established that miRNAs play a role in the regulation of normal hematopoiesis and in HSC maintenance. Similarly, miRNAs regulated the behavior of LSCs, their self-renewal and development [11,54]. In fact, LSCs showed a differential expression of miRNAs with respect to HSC. Therefore, modulation of miRNA aberrantly expressed in LSC and, consequently, the regulation of their targets could determine the elimination of resistant LSCs, by inducing apoptosis or by sensitizing them to chemotherapy [123].

Although many studies reported miRNA profiles of primary AML cell bulk, as described above, little is known about miRNA expression in LSCs.

Recently, de Leeuw et al. compared LSCs (CD34^+^ CD38^−^) versus HSCs, and LSCs versus leukemic progenitors (CD34^+^ CD38^+^), all from the same AML BM, identifying multiple differentially expressed miRNAs. In particular, they found *miR-551b*, *miR-10a*, *miR-151-5p*, *miR-29b*, and *miR-125b* were higher expressed in HSCs than in LSCs, while *miR-181b*, *miR-221*, *miR-21*, and *miR-22* were expressed at lower levels in HSCs than in LSCs [82]. Information about their role in leukemia pathogenesis are reported in Table 2.

It was also reported *miR-126* increases in LSCs and HSC compared to leukemic progenitors, suggesting *miR-126* as a stem cell-associated miRNA [82]. It was found to restrain cell cycle progression, prevent differentiation, and increase self-renewal of LSCs, in vivo, by regulating the phosphatidylinositol-3-kinases/Protein Kinase B/mechanistic target of rapamycin (PI3K/AKT/MTOR) signaling pathway that drives LSC self-renewal and chemotherapy resistance. Interestingly, it also exerted an opposite functional effect in LSCs with respect to HSC; in fact, reduced *miR-126* levels induced HSC expansion in vivo, while impaired LSC maintenance [81]. Finally, *miR-126* inhibition caused apoptosis and decreased clonogenic capacity of LSCs [79,82].

Other altered miRNAs in LSCs were involved in development of leukemia. Wong et al. showed that *miR-17* polycistron maintained LSC potential in a mouse model of MLL-AML by modulating the expression of the cyclin-dependent kinase inhibitor p21 [58]. Moreover, in MLL-AML, the inhibition of *miR-196* and *miR-21* reduced LSCs in vivo [124].

Han et al. reported that *miR-29a* was highly expressed in HSC and primary human LSC, but downregulated in more committed progenitors; this work suggested *miR-29a* role in the regulation of early hematopoiesis and in the initiation of AML by converting myeloid progenitors into self-renewing LSC [125].

Comparing LSCs versus leukemic progenitors, it was found *miR-1274a*, *miR-886*, and *miR-1305* were more lowly expressed in LSCs with respect to leukemic progenitors, while *miR-126-5p*, *miR-126-3p*, *miR-22*, *miR-335*, and *mir-150* were more highly expressed in LSCs than in leukemic progenitors [82].

Interestingly, MVs released by LSC were able to promote proliferation and migration, and to inhibit apoptosis of AML cells. Restoration of *miR-34a* in LSC not only inhibited LSC proliferation, but also generated MVs containing a high level of *miR-34a*, which could revert the effects of LSC-MVs on AML cells [126].

Collectively, the reported studies suggested that miRNA targeting in LSC could be a potential strategy to eradicate leukemic cells. Further studies are necessary to define this aspect.

## 5. miRNAs as Biomarkers in AML

miRNAs have several properties of good biomarkers, such as their presence in various biological fluids (e.g., serum, plasma, urine, saliva, etc.), sequence conservation between human and animal models, and available sensitive measurement methodologies [127].

Moreover, they show high stability and survive in unfavorable conditions, such as extreme variations in pH, boiling, multiple freeze–thaw cycles, and extended storage [128].

The majority of miRNAs are intracellularly localized; however, they are also cell-free: (i) protected into apoptotic bodies and EVs; (ii) associated with proteins of the Argonaute (AGO) family; (iii) joined with high-density lipoprotein [128]. These miRNAs are generally defined as circulating miRNAs. Emerging evidence shows that circulating miRNAs have been gaining importance for their ability to provide early, sensitive, and non-invasive biomarkers in solid and hematologic cancers [129,130,131]. In particular, different studies have identified as candidates specific circulating miRNAs as new biomarkers in AML disease [132,133].

As mentioned above, miRNA expression profile is aberrant in AML and, besides being associated with genetic alterations, hold a diagnostic and prognostic relevance. The evaluation of a single or panel of miRNAs with potential diagnostic/prognostic impact could implement data from cytogenetic, gene mutations, and gene expression profiles.

In Table 3 it’s reported cellular and circulating miRNAs with diagnostic power, selected from the most significant diagnostic studies based on receiver operator characteristic (ROC) curve analysis [134,135,136,137,138,139,140].

In our recent work, we demonstrated that EV *miR-155* was highly expressed in AML with respect to healthy subjects; notably, ROC curve analysis revealed that this miRNA could be a potential new biomarker in AML [139].

We also summarized, in Table 4, miRNAs involved in AML prognosis, reporting their expression level, origin, and their impact in predicting relapse or progression, as well as their relationship with overall survival (OS) and relapse free survival (RFS) [29,33,34,45,46,70,135,136,140,141,142,143,144,145,146,147,148,149,150,151,152,153,154,155,156,157,158,159,160,161,162]

Of note, discovery of new sensitive and specific biomarkers may be clinically useful in MRD monitoring. Notably, circulating miRNAs could be exerting an important relevance in this context, and their quantification could be more accurate over conventional biomarkers. In this setting, Hornick et al. investigated Exo miRNAs as minimally invasive biomarkers in AML. They developed a xenograft model for high-risk AML. In mouse serum Exo, they detected a panel of biomarkers, *miR-150*, *miR-155*, and *miR-1246*, that resulted in equal sensitivity for detection of MRD, when compared to conventional immuno-phenotypic cell evaluation [163].

Despite the appealing use of circulating miRNAs as novel biomarkers, optimization and standardization of purification and quantification are needed to translate basic research into clinical practice. Further studies are required to promote the future use of EV analysis in clinical applications. In particular, accurate procedures concerning sample collection, EV isolation and storage, as well as EV-miRNA quantification, are necessary to improve the use of EVs as biomarkers [127,164].

## 6. miRNAs as Therapeutic Targets

Drug resistance against conventional chemotherapy in AML is one of the major reasons of treatment failure [165]. Current therapies in AML are able to rapidly target dividing blast populations, but have limited capacity to eradicate the functionally distinct LSCs, responsible for disease resistance and relapse [79]. Hence, the identification of new therapeutic strategies in AML is of paramount importance. In this context, miRNA-based therapeutic approaches may provide a novel promising strategy.

The principal approach to target miRNAs was the manipulation of their expression, by the replacing of cancer suppressive miRNAs or by the inhibition of overexpressed oncogenic ones [166]. The replacement therapy employs miRNA mimics (double stranded oligonucleotides) delivered by viral [167] or synthetic vectors, such as lipid-based nanocarriers, amphiphilic star copolymer, inorganic nanoparticles polymeric and dendrimer-based vectors [166,168,169]. These vectors are essential to avoid degradation and to increase cellular uptake of synthetic miRNAs [169].

A consistent number of reports have shown encouraging results of miRNA-based therapeutic approaches, in both in vitro and in vivo studies. For example, *miR-29b*, an AML tumor suppressor [64], was overexpressed in AML blasts, by nanoparticle-based delivery system, decreasing leukemic cell growth and improving the survival of AML-xenograft mouse model [169]. These data were confirmed in another in vivo study in which *miR-29* family member restoration caused a dramatic reduction of AML engraftment in mouse BM, associated with relieved splenomegaly, reduced neoplastic infiltration, decreased cell proliferation, and increased apoptosis activities in spleens [63]. Xu et al., instead, demonstrated that the overexpression of *miR-150* in vitro inhibited proliferation and clonogenic growth, and attenuated tumorigenic activity of LSCs, downregulating the expression of cancer stem cell factors like Nanog, Notch2, and CTNNB1. Moreover, an in vivo study confirmed that *miR-150* overexpression progressively abrogated tumor growth [89]. The inhibition of leukemia progression was also demonstrated by forced expression of *miR-22* and *miR-193a*. In particular, Jiang et al. demonstrated that the overexpression of *miR-22*, by nanoparticles, significantly suppressed leukemic cell viability and growth in vitro, and repressed leukemia development and maintenance in vivo [61]. Instead, Li et al. showed that *miR-193a* restoration by synthetic miRNA contributed to the blockage of malignant-cell proliferation in t(8;21) AML xenograft model [99]. Moreover, Yang et al. described *miR-122* as tumor suppressor miRNA in childhood AML, and showed that a *miR-122* mimic efficiently inhibited cell proliferation and cell cycle of HL-60 AML cell line [53]. Another potential candidate for miRNA therapy in AML is *miR-34*. Wang et al. showed that overexpression of this miRNA in AML cell lines reduced both mRNA and cell surface protein expression of PD-L1, an inhibitory immune checkpoint molecule, suggesting the potential role of *miR-34a* mimic also in AML immunotherapy [68].

The second miRNA-based therapeutic approach is the silencing of aberrantly expressed miRNAs using anti-miRNA oligonucleotides (AMOs), miRNA sponges, or miRNA masking [170]. These synthetic oligonucleotides have sequences complementary to endogenous mature miRNA, and competitively prevent the interaction between miRNAs and their targets [166]. *MiR-126*, for example, high expressed in CN-AML, was silenced in in vivo models, causing a reduction of LSCs and showing no toxic effect on normal hematopoietic functions. In this study, Dorrance et al. identified a potential novel therapeutic approach to treat AML patients and prevent relapse [79]. Literature data also indicated that *miR-181* inhibition could provide a new strategy for AML therapy [171], increasing the differentiation of myeloid progenitors, reducing the engraftment and the infiltration of leukemic HSPCs into BM and spleen, and ameliorating symptoms of leukemia in AML CD34^+^ HSPC xenograft mice [171]. Another miRNA involved in leukemogenesis seemed to be *miR-9*; Tian et al. demonstrated that its knockdown in vivo suppressed malignant cell proliferation, decreased leukemic cell counts both in blood and BM, and reduced splenomegaly [172]. Velu et al., instead, studied the combination of *miR-21* and *miR-196b* silencing in murine models of MLL-AF9 leukemia [124]. They demonstrated that co-treatment with both antagomirs improved the outcome of MLL-AF9 leukemia, and did not show toxicity in vivo. In this study, they tested both antagomir with or without a cholesterol modification, demonstrating highest efficacy of anti-miRNAs with lipoprotein particles (the commercial ones) in vivo [124].

Emerging evidence revealed that modification of miRNA expression levels can increase the sensitivity to chemotherapy or other drugs. In this context, Chen et al. demonstrated that overexpression of *let-7a*, in xenograft mouse of primary human AML cells, increased the cytarabine (Ara-C) efficacy respect to single chemotherapeutic treatment and extended survival of mice [173]. Also, the overexpression of *miR-150* in vitro reduced cell viability of two AML cell lines treated with Ara-C [89]. Moreover, Lu et al. reported that, also, forced *miR-181b* expression rendered leukemic cells more sensitive to doxorubicin and Ara-C in vitro [174]. Gao et al., instead, demonstrated a synergistic apoptotic effect of 5-azacytidine and *miR-193a* mimic combination in AML cell line, Kasumi-1, suggesting *miR-193a* restoration as a potential adjuvant therapy in c-KIT-positive AML [100]. In a recent study, it was also demonstrated that the upregulation of *miR-217*, a tumor suppressor in AML, induced in vitro chemosensitivity to doxorubicin [165]. Additionally, it was reported that ectopic expression of *miR-133*, a tumor suppressor for EVI1-overexpressing AML cells, increased drug sensitivity specifically in this subset of AML [175].

Instead, Lechman et al. suggested *miR-126* inhibition as a strategy to overcome LSC chemoresistance, and to increase the sensitivity of anti-proliferative drugs [81]. Furthermore, targeting of *miR-21* and *miR-196b* increased the effect of induction chemotherapy in xenograft model of MLL leukemia [124].

Another interesting concept concerns the effects of existing drugs on miRNA expression. For example, lenalidomide, a drug approved for the treatment of multiple myeloma and MDS, induced endogenous expression of *miR-181* with significant tumor growth inhibition in AML xenograft mouse models [176]. Additionally, Khalife et al. reported that MLN4924 drug (Pevonedistat), decreasing NF-κB activation, downregulated oncogenic *miR-155* levels in FLT3-ITD^+^ AML cell lines and reduced leukemic phenotypes, in both in vitro and in vivo models [177].

The identification of miRNA target genes can help to understand the multiple mechanisms involved in AML [165], and can represent another important tool to provide targeted AML treatment strategies [166]. For example, miRNA activity can be regulated by blocking its access to the target mRNA. A recent study showed that the activity of two oncomiRs, *miR-9* and *miR-17*, could be antagonized by the induction of two RNA binding proteins, RBM38 and DND1, whose function is to protect mRNAs from miRNA-mediated cleavage [178].

MVs represent another important tumor suppressive miRNA vehicle. In a recent work, it was hypothesized that miRNA contents of LSC-derived MVs (LMVs) can be reprogrammed, altering the effects of LMVs on cancer cells [126]. In particular, MVs isolated from human AML cell line KG-la, previously transfected with *miR-34a* mimic, were used to inhibit proliferation and migration of AML cell by the modulation of caspase-3 and Tim-3 levels [126].

Despite the above described miRNA-therapeutic approach advantages, there are still many challenges to be solved before introducing it into the clinic. Furthermore, although the literature suggests limited toxicity of some of these miRNAs in various model systems, clinical trials are necessary to evaluate whether this preclinical promise will be recapitulated in human patients. Likewise, concentration and dose plans need to be evaluated for efficacy and toxicity, and the long-term effects following miRNA therapy must be assessed. Additionally, as the therapeutic potential of different miRNAs has been investigated, the combination of multiple miRNAs could be evaluated as a possible miRNA therapy in AML.

Nevertheless, a major limitation preventing the development of miRNA-based therapies is the difficulty to deliver drugs to BM, and the necessity of using higher doses to elicit a therapeutic effect [14].

## 7. Long Non-Coding RNAs Can Interfere with miRNA Function in AML

Although a broad range of non-coding RNAs has been discovered, miRNAs and long non-coding RNAs (lncRNAs) are the best studied. LncRNAs are RNAs longer than 200 nucleotides. Based on the genomic locations where lncRNAs are transcribed, they can be classified into sense, overlapping with at least part of another gene in the same strand; antisense, overlapping with at least part of another gene on the opposite strand; intronic, originating from the intron of another gene; intergenic, not overlapping with any gene; and chimeric, resulting from fusion products due to chromosomal rearrangements [179].

LncRNAs could promote the strength of specific enhancer–promoter looping, contributing to gene activation [179].

Their expression is tightly controlled, and exhibits higher cell specificity than proteins, including lineage-determining transcription factors [180]. LncRNAs play an important role in several biological processes, including cell development, differentiation, proliferation, invasion, and migration [181]. LncRNAs regulate gene expression at the epigenetic, transcriptional, and post-transcriptional level [182].

Aberrant expression of miRNAs and lncRNAs has been described in all types of cancers [183,184].

In AML, aberrant expression of lncRNAs contributes to initiation, maintenance, and development of leukemogenesis [179]. They are involved in chromatin remodeling, as well as transcriptional and post-transcriptional regulation, through a variety of chromatin-based mechanisms and via cross-talk with other RNA species. LncRNAs can function as decoys, scaffolds, and enhancer RNAs [185]. Moreover, lncRNAs competed with endogenous miRNAs in AML.

A lncRNA, *CCAT1*, for example, was found overexpressed in AML; it repressed monocytic differentiation, and promoted AML cell growth by sequestering *miR-155* [186]. In accord with Palma’s study [107], Chen’s work showed that *miR-155* has a pro-differentiation and anti-proliferation role in FLT3 wild-type AML cells [186], in contrast to its oncogenic function reported in FLT3-ITD mutated AML [91,187]. These data suggested that the gene regulation by *miR-155* is complex, as it targets both tumor suppressor genes or oncogenes, and its function in a specific subtype of AML is likely subject to disease context and cell type [107,186].

An oncogenic activity of lncRNA was also shown by *HOTAIR* that competitively binds *miR-193a*, an important tumor-suppressor miRNA, thus modulating the expression of c-Kit in AML cells [188]. The lncRNA *HOTAIRM1*, instead, was associated with myeloid differentiation; it also had pivotal roles in the degradation of oncoprotein PML-RARA, by acting as a microRNA sponge, sequestering *miR-20a*, *miR-106b*, and *miR-125b*, whose targets are autophagy-associated genes [189].

Moreover, Mangiavacchi et al. showed that the host non-coding transcript of *miR-223*, *linc-223*, is a functional lncRNA that controls proliferation and differentiation of AML cells by binding *miR-125-5p*. This lncRNA is an oncogenic miRNA upregulated in primary AML where *linc-223* was instead downregulated [190].

In conclusion, lncRNA functions introduce an extra layer of complexity in the miRNA-target network, making it necessary to study the interactions between miRNAs and lncRNAs, both to shed light on AML pathogenesis and on therapy.

## 8. Conclusions

miRNAs have emerged as a class of gene expression pivotal regulators contributing to AML pathogenesis and as potential biomarkers. Actually, miRNA analysis has not yet been included in AML clinical practice. Specific miRNA expression could help clinicians to classify subtypes, to determine prognosis, and to predict the response of treatment in AML.

Particular attention should be given to circulating miRNA, both free or contained in EVs, which are easy to collect with a non-invasive peripheral blood sample. In addition, while malignant cells are usually reduced after treatment, circulating RNAs can still be detected. Finally, miRNA analysis, through advanced next generation sequencing, will provide more details on miRNAs, and also lncRNAs involved in leukemia onset and progression. This information supports the concept that circulating miRNAs, in addition to current clinical AML parameters, could have significant value as new AML biomarkers for detection of disease progression. They can also serve as indicators of therapeutic response, and in MRD detection in a future clinical practice.

Another approach to improving AML outcomes could be the potential use of miRNAs as therapeutic targets. miRNA modulation could be obtained through new strategies by using miRNA inhibitors or mimics.

Moreover, miRNA therapy in combination with current chemotherapy could potentially eradicate LSCs.

Actually, the main problems in developing miRNA therapy are the identification of the best miRNA candidates and the design of miRNA delivery vehicles that confer higher stability to the therapeutic candidate, avoiding potential toxicities and off target effects [191]. Many complexities and mysteries remain in miRNA biology; miRNA studies will be key to our understanding of how to improve their use in an AML clinical setting.

**Figure 1:**
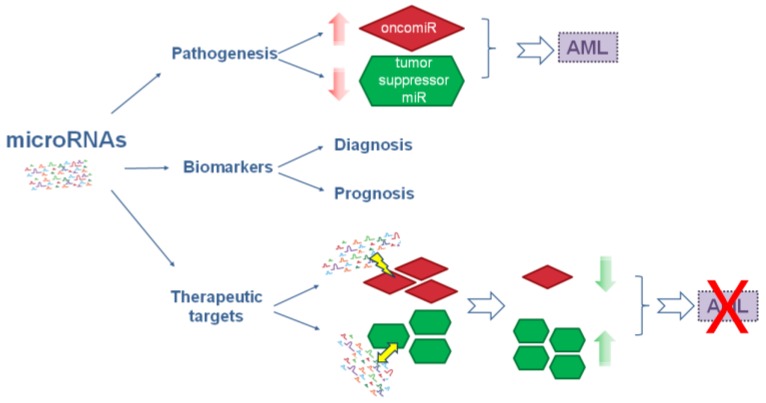
Schematic diagram of microRNAs role in AML. MicroRNAs are involved in pathogenesis and are considered as biomarkers and therapeutic targets in AML. Red arrows indicate overexpression of oncomiR and downexpression of tumor suppressor miR which cause AML; lightning and two-headed arrow indicate targeting and restore, respectively; green arrows indicate decreased oncomiR or increased tumor suppressor miR levels that block AML; red cross indicates AML block.

The role of miRNAs in AML is reported in a schematic diagram in Figure 1.

## Figures and Tables

**Table 1:** MicroRNA (miRNA) expression in acute myeloid leukemias (AML) with recurrent genetic abnormalities.

Genetic Abnormalities	miRNAs			
	Upregulated		Downregulated	
t(8;21)(q22;q22.1) RUNX1-RUNX1T1	*miR-126*, *miR-320*, *miR-328*, *miR-449b*;	[22]	*miR-9*, *miR-10a*, *miR-125b*, *miR-133a*, *miR-135a*, *miR-148a*, *miR-196a*, *miR-221*;	[22]
	miR-126/126*;	[23]	*miR-133a*;	[24]
	*miR-146a*;	[24]	*let-7b*, *let-7c*;	[26]
	*miR-146a*, *miR-181a*, *miR-181b*	[25]	*miR-221*, *miR-222*, *miR-223*;	[27]
			*miR-223*;	[28]
inv(16)(p13.1q22) or t(16;16)(p13.1;q22) CBFB-MYH11	miR-126/126*;	[23]	*miR-29c*, *miR-149*;	[22]
	*miR-99a*, *miR-100*, *miR-224*;	[24]	*miR-92*;	[23]
	*miR-212*	[29]	*miR-127*, *let-7b*, *let-7c*;	[26]
			*miR-221*, *miR-222*, *miR-223*	[27]
PML-RARA	*miR-100*, *miR-181a*, *miR-181b*, *miR-181c*, *miR-181d*, *miR-224*, *miR-368*, *miR-382*, *miR-424*;	[23]	*miR-17-5p*, *miR-20a*, miR-126/126*, *miR-150*;	[23]
	*miR-127*, *miR-154*, miR-154*, *miR-299*, *miR-323*, *miR-368*, *miR-370*;	[24]	*miR-17-3p*, *miR-185*, *miR-187*, *miR-194*, *miR-200a*, *miR-200b*, *miR-200c*, *miR-330*, *miR-339*;	[24]
	*miR-100*, *miR-125b*, *miR-146-5p*, *miR-181a*, *miR-181b*;	[25]	*miR-18b*, *miR-22*, *miR-24*, *miR-27a*, *miR-126-3p*, *miR-150*, *miR-223*, *miR-342-3p*, *miR-378 let-7b*, *let-7c*;	[25]
	*miR-127*, *miR-134*, *miR-299-5p*, *miR-323*, *miR-376a*, *miR-382*;	[26]	*miR-107, miR-342, let-7c*	[30]
	*miR-15a*, *miR-15b*, *miR-16*, *miR-142-3p*, *miR-142-5p*, *miR-223*, *let-7a*, *let-7d*;	[30]		
	*miR-128a*	[31]		
t(9;11)(p21.3;q23.3) MLLT3-KMT2A	*miR-196b*;	[32]	*miR-29a*, *miR-29b*, *miR-29c*;	[34]
	*miR-196b*;	[33]	*miR-495*	[35]
	*miR-326*	[34]		
t(6;9)(p23;q34.1);DEK-NUP214	-		-	
inv(3)(q21.3q26.2) or t(3;3)(q21.3;q26.2); GATA2, MECOM	-		*miR-30a-3p*, *miR-95*, *miR-101*, *miR-145*, *miR-199b*, *miR-422a miR-618*	[22]
t(1;22)(p13.3;q13.3) RBM15-MKL1	-		-	
BCR-ABL1	-		-	
mutated NPM1	*miR-10a*, *miR-10b*, *miR-196a*, *miR-196b*;	[26]	*miR-22*, *miR-127*, *miR-128a*, *miR-135a*, *miR-139*, *miR-145*, *miR-192*, *miR-193b*, *miR-198*, *miR-204*, *miR-299*, *miR-324*, *miR-326*, *miR-373*, miR-373*, *miR-383*, *miR-429*, *miR-486*, *miR-493*, *miR-498*;	[36]
	*miR-9*, *miR-10a*, *miR-10b*, *miR-15a*, *miR-16a*, *miR-16b*, *miR-16-1*, *miR-16-2*, *miR-17*, *miR-18a*, *miR-19a*, *miR-19b*, *miR-20*, *miR-21*, *miR-24*, *miR-29a*, *miR-29b*, *miR-29c*, *miR-100*, *miR-98*, *miR-102*, *miR-106*, *miR-142*, *miR-152*, *miR-155*, *miR-195*, *miR-369*, *miR-378*, *miR-374*, *let-7a-1*, *let-7a-2*, *let-7a-3*, *let-7c*, *let-7d*, *let-7f*, *let-7g*;	[36]	*miR-126*, *miR-130a*, *miR-424*, *miR-450a*, *miR-451*, *miR-486-5p*;	[37]
	*miR-9*, *miR-10a*, *miR-10b*, *miR-100*, *miR-196a*, *miR-196b*, *let-7a*, *let7c*;	[37]	*miR-192*, *miR-495*;	[38]
	*miR-7*, *miR-10a*, *miR-10b*, *miR-15a*, *miR-15b*, *miR-16*, *miR-17*, *miR-18a*, *miR-28-5p*, *miR-19a*, *miR-19b*, *miR-20a*, *miR-21*, *miR-23a*, *miR-23b*, *miR-27a*, *miR-27b*, *miR-29a*, *miR-29b*, *miR-29c*, *miR-30a*, *miR-30b*, *miR-30c*, *miR-30e*, *miR-92a*, *miR-98*, *miR-105*, *miR-106a*, *miR-124*, *miR-125a-5p*, *miR-125b*, *miR-136*, *miR-142-5p*, *miR-146a*, *miR-151-5p*, *miR-155*, *miR-195*, *miR-196b*, *miR-199a-3p*, *miR-200b*, *miR-221*, *miR-223*, *miR-224*, *miR-320*, *miR-339-5p*, miR-340*, *miR-361-5p*, *miR-369-3p*, *miR-373*, *miR-379*, *miR-380*, *miR-381*, *miR-382*, *miR-491-5p*, miR-493*, *miR-494*, miR-518c*, *miR-526b*, *let-7a*, *let-7b*, *let-7c*, *let-7d*, *let-7e*, *let-7f*	[38]	*miR-424*	[39]
biallelic mutations of CEBPA	*miR-128*, *miR-181a*, miR-181a*, *miR-181b*, *miR-181c*, *miR-181d*, *miR-192*, *miR-219-1-3p*, *miR-224*, *miR-335*, *miR-340*	[40]	*miR-34a*, *miR-194*;	[40]
			*miR-34a*	[41]
mutated RUNX1	*miR-21*, *miR-220*, *miR-595*	[42]	*miR-99a*, *miR-100*, *miR-223*, *let-7a*, *let-7f*	[42]
FLT3-ITD	*miR-10b*, *miR-155*;	[26]	*miR-144*, *miR-451*, *miR-486-5p*, *miR-488*	[43]
	*miR-128a*;	[31]		
	*miR-10a*, *miR-10b*, *miR-155*;	[34]		
	*miR-155*;	[36]		
	*miR-155*;	[39]		
	miR-125b2*;	[43]		

*: complementary strand to microRNA.

**Table 2:** miRNAs involved in AML pathogenesis.

miRNA	Altered Expression	Mechanism of Dysregulation	Targets	Functional Effect of Mirna Altered Expression	Refs
*miR-9*	Up: in MLL-AML	Promoter targeted by MLL-fusion proteins	RHOH, RYBP	Increased proliferation, survival and leukemogenesis in mice	[55]
	Down: in t(8;21) AML		HMGA2, LIN28B	Increased proliferation and decreased monocytic differentiation	[56]
	Down: in EVI1-induced AML	EVI1 hypermethylates promoter	FOXO1, FOXO3	Increased proliferation and decreased monocytic differentiation	[57]
*miR-17-92* cluster	Up: in LSCs in MLL-AML	Activated by MYC	P21	Increased proliferation, survival, differentiation, self-renewal, colony forming capacity and leukemogenesis in mice	[58]
		Genomic amplification and upregulation by MLL-fusion proteins		Inhibited differentiation and apoptosis, promoted cell proliferation	[59]
*miR-22*	Up: in MDS/MDS-derived AML		TET2	Increased proliferation, survival, self-renewal and decreased differentiation. *miR-22* overexpression led to myeloid malignancy in mice	[60]
	Down: in de novo AML	Downregulated via TET1/GFI1/EZH2/SIN3A-mediated epigenetic repression and DNA copy-number loss	CRTC1, FLT3, MYCBP	Increased AML blast cell growth. Decreased differentiation and increased leukemic progression in mice	[61]
	Down: in de novo AML	Increased with loss of PU.1	MECOM	Increased AML blast cell growth. Decreased differentiation and increased leukemic progression	[62]
*miR-29b*	Down: in various subtypes of AML	Repressed by c-MYC	AKT2, CCND2	Increased cell growth, leukemic progression in vivo	[63]
	Down: in various subtypes of AML		MCL-1, CXXC6, CDK6	Increased cell growth, decreased apoptosis, leukemic progression in vivo	[64]
	Down: in t(8;21) AML	Repressed by MYC and NF-κB	SP1	KIT upregulation contributing to malignant proliferation	[65]
	Down: in CEBPA mutated AML	Downregulated via loss of CEBPA; chromosome 7q deletions			[66]
	Down: in various subtypes of AML		SP1, DNMT3A, DNMT3B	*miR-29b* downregulation led global DNA hypermethylation	[75]
*miR-34a*	Down: in CEBPA mutated AML	Downregulated via loss of CEBPA	E2F3	Increased proliferation and decreased differentiation	[41]
	Down: in de novo AML	Downregulated via loss of MUC1	PDL1	Immune dysregulation	[67]
	Down: in de novo AML		PDL1	Immune dysregulation	[68]
	Down: in CEBPA mutated AML cell lines		HMGB1	Inhibited cell apoptosis and increased autophagy	[69]
*miR-99*	Up: in initial diagnosis and relapse			Regulated self-renewal, inhibiting differentiation and cell cycle entry	[70]
	Up: in AML-AF9		SMARCA5, HS2ST3, HOXA1	Increased proliferation, colony formation, cell survival, inhibited differentiation	[71]
	Up: in pediatric-onset AML (M1–M5)		CTDSPL TRIB2	Increased proliferation, colony formation, cell survival	[72]
*miR-125b*	Up: in MDS and AML with t(2;11)(p21;q23)	Increased by t(2;11)(p21;q23)		Inhibited differentiation	[73]
	Up: in AML		LIN28A	Uncontrolled generation of myeloid cells	[74]
				*miR-125b* overexpression led to AML in mice	[76]
			IRF4	*miR-125b* overexpression induced myeloid leukemia in mice by inducing immortality, self-renewal and tumorigenesis in myeloid progenitors	[77]
	Up: in pediatric AML			*miR-125b* was associated with PML/RARA status	[78]
			FES, PU.1	Blocked monocytic differentiation of AML in vitro	[105]
*miR-126*	Up: in t(8;21) and inv(16) AML	Epigenetic regulation	PLK2	Inhibited cell apoptosis and increased cell viability	[23]
	Up: in LSC of CN-AML	Epigenetic regulation		Increased LSC maintenance and self-renewal	[79]
	Up: in t(8;21) AML		ERRFI1, SPRED1, FZD7	Both gain and loss of function of *miR-126* promoted leukemogenesis in vivo through targeting distinct gene signaling	[80]
	Up: in LSCs of AML	Epigenetic regulation	ADAM9, ILK, GOLPH3, CDK3, TOM1	Increased LSC maintenance and self-renewal, quiescence, chemotherapy resistance in vivo	[81]
	Up: in LSCs of AML			Increased leukemic growth, and survival of leukemic stem and progenitor cells in vivo	[82]
*miR-128*	Up: in AML LSC, highest in FLT3-ITD and PML-RARalfa		LIN28A	Increased proliferation and decreased differentiation	[31]
*miR-145 miR-146a*	Down: in AML with normal karyotype			Resulted in AML in vivo	[83]
*miR-146a*	Down: in del(5q) MDS		TIRAP, TRAF6	Inappropriate activation of innate immune signaling in HSPCs and megakaryocytic abnormalities	[84]
	Deleted: in del(5q) MDS/AML	Deletion in del(5q) MDS/AML		Co-deletion of TIFAB and *miR-146a* may cooperate to induce TRAF6 signaling contributing to ineffective hematopoiesis	[85]
			IRAK1	*miR-146a* knockout mice developed myeloid and lymphoid malignancies	[86]
	Deleted: in del(5q) MDS/AML	Deletion in del(5q) MDS/AML		Increased cell survival and proliferation of propagating cells through the TRAF6/p62/NF-κB complex	[87]
				*miR-146a* deletion led to myeloproliferation in mice	[88]
*miR-150*	Down: in various subtypes of AML		NANOG	Increased proliferation, colony and sphere formation, increased tumor growth in vivo	[89]
	Down: in various subtypes of AML		EIF4B, FOXO4, PRKCA, TET3	Increased cell growth and inhibited apoptosis in vitro and in vivo	[90]
*miR-155*	Up: in FLT3-ITD+ AML	Targeted by STAT5 and NF-κB	PU.1	Inhibited myeloid differentiation. Proliferation and survival of FLT3-ITD leukemic cells	[91]
	Up: in CN-AML			Negative impact on outcome	[92]
	Up: in MLL AML	Upregulated by MLL fusion genes via MEIS1		No effect in MLL-AF9 mouse model	[93]
	Up: in FLT3-ITD+ AML		CEBPB, SHIP1	*miR-155* overexpression led to myeloproliferative neoplasm in mice	[94]
	Up: in AML (FAB M4-M5)			Enforced expression of *miR-155* in HSCs caused a myeloproliferative disorder in mice BM	[95]
	Up: in FLT3-ITD+ AML	Upregulated by IL3			[96]
	Up: in various subtypes of AML	Epigenetically regulated	PU.1	Leukemic cell growth of blast cells	
*miR-182*	Down: in AML with CEBPA mutation, t(8;21) and t(15;17)	Upregulated via loss of CEBPA	CEBPA	Decreased myeloid differentiation	[97]
*miR-192*	Down: in various subtype of AML		CCNT2	Increased proliferation and cell cycling, decreased differentiation	[98]
*miR-193a*	Down: in t(8;21) AML	Epigenetically silenced by AML1/ETO	AML1/ETO, DNMT3A, HDAC3, KIT, CCND1, MDM2	Decreased apoptosis and differentiation	[99]
	Down: in various subtypes of AML	Epigenetically repressed by promoter hypermethylation	KIT	Increased cell growth. decreased apoptosis and differentiation, and increased KIT expression	[100]
*miR-194-5p*	Down: in AML cell line	Increased by HDACi SAHA treatment in AML cells	BCLAF1	Decreased apoptosis, differentiation	[101]
*miR-196b*	Up: in MLL-associated AML	Activated by MLL-fusion proteins		Increased proliferation and survival. Decreased differentiation and replating potential.	[32]
	Up: in MLL-associated AML	Activated by MLL-fusion proteins; Co-expressed with HOXA9 in MLL rearranged leukemia	HOXA9, MEIS1, FAS	Inhibited differentiation, promoted cell proliferation via inhibiting apoptosis. Induced leukemic progression in mice	[33]
*miR-223*	Down: in t(8;21) AML	Epigenetically silenced by AML1/ETO		Myeloid differentiation block	[28]
	Down: in various subtypes of AML	Activated by CEBPA and repressed by E2F1 transcription factors	E2F1	Increased proliferation. Decreased differentiation/granulopoiesis	[102]
	Down: in various subtypes of AML		FBXW7	Increased cell proliferation and enhanced apoptosis	[103]
	Down: in AML with adverse prognosis			Impaired differentiation	[104]

Abbreviations: AML, acute myeloid leukemia; LSCs, leukemia stem cells; MDS, myelodysplastic syndrome; CN-AML, cytogenetically normal AML.

**Table 3:** miRNAs as diagnostic biomarkers in AML.

miRNA	Expression	Specimen	Refs
*miR-29a*, *miR-142-3p*	under-expression	PBMCs	[134]
circulating *miR-10a-5p*	over-expression	serum	[135]
circulating *miR-10a-5p*, *miR-93-5p*, *miR-129-5p*, *miR-155-5p*, *miR-181b-5p*, *miR-320d*	over-expression	serum	[136]
circulating *miR-92a*, *miR-143*, *miR-342*	under-expression	plasma	[137]
circulating *miR-150*, *miR-342*	under-expression	plasma	[138]
circulating *miR-155*	over-expression	serum EVs	[139]
circulating *miR-370*	under-expression	serum	[140]

Abbreviations: PBMCs, peripheral blood mononuclear cells; EVs, extracellular vesicles.

**Table 4:** miRNAs as prognostic biomarkers in AML.

miRNA	Expression	Prognostic Impact	Specimen	Refs
*miR-9*	overexpression	unfavorable OS and RFS	BMMCs	[141]
*miR-24*	overexpression	shorter OS	BMMCs/PBMCs	[142]
*miR-26a*, *miR-29b*, *miR-146a*	overexpression	shorter OS	BMMCs	[143]
*miR-29a*	underexpression	shorter OS and RFS	BMMCs	[144]
*miR-29b*	underexpression	poorer OS	BMMCs/PBMCs	[145]
*miR-96*	underexpression	lower OS and RFS	BMMCs/PBMCs	[146]
*miR-99a*	overexpression	worse OS and EFS	BMMCs	[70]
*miR-124-1*	underexpression	longer OS RFS	BMMCs	[147]
*miR-135a*, *miR-409-3p*	underexpression	higher cumulative incidence of relapse	BMMCs/PBMCs	[148]
*miR-181a*, *miR-181b*	overexpression	decreased risk of an event (i.e., lack of complete remission, relapse, or death)	BMMCs	[45]
*miR-181a*	overexpression	favorable prognosis	BMMCs	[149]
*miR-181a*, *miR-181b*, *miR-181d*	overexpression	longer OS	not specified	[46]
*miR-181a*	overexpression	higher CR rate, longer OS	BMMCs	[150]
*miR-181b*	overexpression	lower CR rates, shorter RFS and OS	BMMCs	[151]
*miR-181b*	overexpression	better prognosis and lower probability of relapse	BMMCs	[152]
*miR-188-5p*	underexpression	longer OS and EFS	not specified	[153]
*miR-191*, *miR-199a*	overexpression	worse prognosis (worse overall and event free survival)	BMMCs	[34]
*miR-196b*	overexpression	shorter OS	leukemic blasts	[33]
*miR-196b*, *miR-644*	overexpression	shorter OS	BMMCs/PBMCs	[148]
*miR-212*	overexpression	better OS, higher CR rate, better EFS and RFS	BMMCs/PBMCs	[29]
*miR-328*	underexpression	poor OS and shorter RFS	plasma	[154]
*miR-331*	overexpression	worse response to therapy and shorter OS	BMMCs	[155]
*miR-375*	overexpression	shorter RFS and OS	BMMCs	[156]
*miR-378*	overexpression	shorter RFS	BMMCs	[157]
*miR-3151*	overexpression	shorter DFS and OS	PBMCs	[158]
*let-7a-3*	overexpression	shorter OS and RFS	BMMCs	[159]
*let-7a-2-3p*	overexpression	longer OS and EFS	not specified	[153]
circulating *miR-10a-5p*	overexpression	shorter OS	serum	[135]
circulating *miR-181b-5p*	over-expression	worse OS	serum	[136]
circulating *miR-183*	overexpression	shorter OS and RFS	serum	[160]
circulating *miR-210*	overexpression	shorter OS and RFS	serum	[161]
circulating *miR-335*	overexpression	shorter OS and RFS	serum	[162]
circulating *miR-370*	underexpression	shorter RFS and OS	serum	[140]

Abbreviations: BMMCs, bone marrow mononuclear cells; PBMCs, peripheral blood mononuclear cells; OS, overall survival; RFS: relapse free survival; EFS: event free survival; DFS, disease free survival; CR: complete remission.
